# Survival in bladder and upper urinary tract cancers in Finland and Sweden through 50 years

**DOI:** 10.1371/journal.pone.0261124

**Published:** 2022-01-04

**Authors:** Kari Hemminki, Asta Försti, Akseli Hemminki, Börje Ljungberg, Otto Hemminki

**Affiliations:** 1 Biomedical Center, Faculty of Medicine, Charles University in Pilsen, Pilsen, Czech Republic; 2 Division of Cancer Epidemiology, German Cancer Research Center (DKFZ), Im Neuenheimer Feld, Heidelberg, Germany; 3 Hopp Children’s Cancer Center (KiTZ), Heidelberg, Germany; 4 Division of Pediatric Neurooncology, German Cancer Research Center (DKFZ), German Cancer Consortium (DKTK), Heidelberg, Germany; 5 Cancer Gene Therapy Group, Translational Immunology Research Program, University of Helsinki, Helsinki, Finland; 6 Comprehensive Cancer Center, Helsinki University Hospital, Helsinki, Finland; 7 Department of surgical and perioperative sciences, Urology and andrology, Umeå University, Umeå, Sweden; 8 Department of Urology, Helsinki University Hospital and University of Helsinki, Helsinki, Finland; 9 Division of Urologic Oncology, Department of Surgical Oncology, Princess Margaret Cancer Center, University Health Network and University of Toronto, Toronto, Ontario, Canada; Universitat des Saarlandes, GERMANY

## Abstract

Survival has improved in bladder cancer but few studies have considered extended periods or covered populations for which medical care is essentially free of charge. We analyzed survival in urothelial cancer (UC, of which vast majority are bladder cancers) in Finland and Sweden over a 50-year period (1967–2016) using data from the NORDCAN database. Finland and Sweden are neighboring countries with largely similar health care systems but higher economic resources and health care expenditure in Sweden. We present results on 1- and 5-year relative survival rates, and additionally provide a novel measure, the difference between 1- and 5-year relative survival, indicating how well survival was maintained between these two periods. Over the 50-year period the median diagnostic age has increased by several years and the incidence in the very old patients has increased vastly. Relative 1- year survival rates increased until early 1990s in both countries, and with minor gains later reaching about 90% in men and 85% in women. Although 5-year survival also developed favorably until early 1990s, subsequent gains were small. Over time, age specific differences in male 1-year survival narrowed but remained wide in 5-year survival. For women, age differences were larger than for men. The limitations of the study were lack of information on treatment and stage. In conclusion, challenges are to improve 5-year survival, to reduce the gender gap and to target specific care to the most common patient group, those of 70 years at diagnosis. The most effective methods to achieve survival gains are to target control of tobacco use, emphasis on early diagnosis with prompt action at hematuria, upfront curative treatment and awareness of high relapse requiring regular cystoscopy follow up.

## Introduction

The vast majority of urothelial cancers (UCs) are located in the bladder (90–95% of all) while the upper urinary tract (UUT) is the second most common location. Here 2/3 are located in the renal pelvis and the remainder in the ureter [1]. Even though UCs constitute a clinical entity, bladder cancers are often studied separately because of their numerical dominance. Bladder cancer is characterized by male excess, ranging from 3- to 6-fold, and by international incidence trends that are correlated with smoking prevalence [2]. Between years 1990 and 2016 the global incidence of and mortality due to bladder cancer have declined modestly [3]. It has been estimated that 27% of global bladder cancer deaths were due to smoking and 7% to high fasting glucose (type 2 diabetes) in 2016 [4]. According to a UK estimated for year 2015, smoking accounted for 46.0% of male bladder cancers and 41.5% female bladder cancers; the only other large contribution was occupational exposures, 7.1% for men [5]. UUT cancers show also male excess and share risk factors with bladder cancer, including smoking, occupational exposures, family history and association with Lynch syndrome [1, 6–9]. However, Lynch syndrome may be a relatively more important risk factor for UUT cancers than for bladder cancer [10, 11].

Surgery has traditionally been the main treatment mode for urothelial cancers. The treatment options for bladder cancer largely depend on how advanced the cancer is, distinguishing early stage, non-muscle-invasive bladder cancer, and more advanced muscle-invasive bladder cancer. Some 20–25% of patients present with muscle-invasive tumors and the remaining patients have superficial tumors, which can later progress to invasive cancer [12]. Non-muscle- invasive tumors are transuretherally resected while muscle-invasive tumors are typically treated with cystectomy; both of which can be supplemented with chemotherapy or immunotherapy [12, 13]. However, trimodal therapy (i.e., transurethral resection, followed by concurrent chemotherapy and radiation) has become an alternative for radical cystectomy in muscle-invasive bladder cancer and has been integrated into treatment options [14]. For UUT cancers treatment may involve removal of the ipsilateral ureter and kidney [1]. Age-standardized relative 5-year survival in bladder cancer in Europe was 68% in period 2000 to 2007 [15]. Survival was 74% for Finnish and Swedish men in 1999–2003 while for women it was 72 and 68%, respectively [16]. Early detection, novel imaging technologies and improvements in treatment have been assumed to contribute to positive trends in bladder cancer survival [13]. A Swedish study reported a strong association of survival and higher educational level in both genders, the results being even more prominent in bladder cancer than in lung cancer [17]. International survival studies have faced problems of variable diagnostic definition in bladder cancer [18]. For UUT cancers prognosis is worse than for bladder cancer [1]. However, the prognosis is comparable at identical stages [19].

Here we assessed UC survival in Finland and Sweden over a period of 50 years. The importance of extended periods of reliable epidemiological data was recently emphasized as a tool of interpreting the underlying ‘signature of cancer’ [20]. Medical care has been practically free-of-charge to the population in these countries, and this with the long follow-up time will offer a unique ‘real world’ perspective on survival in UC. In analyzing differences between 1-year and 5-year survival and age group specific survival we try to understand factors that have contributed or hampered favorable development in survival.

## Materials and methods

We considered UCs diagnosed from 1967 to 2016 identified from the Nordcan database which is a compilation of data from the high-level Nordic cancer registries as described [21] (https://NORDCAN.iarc.fr/en/database#bloc2). The used codes by NORDCAN were C65-68 (cancers of the pelvis, ureter, bladder), D09.0–1, D30.1–9, D41.1–9 (in situ and tumors of undefined behavior at these sites). Incidence data were adjusted to the world standard population. In assessing incidence trends, estimated annual percentage change (EAPC) was used to describe the magnitude of change in the trend on fitting a regression model to the log of the age standardized incidence rate. This described the average annual rate change (%) over the time period selected.

All survival data are ‘relative survival’ which is defined as the ratio of the observed survival in the group of patients compared to the survival expected in the general population, adjusted for sex, age and calendar time at the time of diagnosis. Survival data were available from 1967 onwards and the analysis was based on the cohort survival method for the first nine 5-year periods from 1964–2011, and a hybrid analysis combining period and cohort survival in the last period 2012–2016, as detailed [22, 23]. The Finnish and Swedish life tables were used to calculate the expected survival.

We calculated also a difference in survival percent between year 1 and year 5 as a measure on how well survival is maintained between years 1 and 5. A small difference indicates high survival between years 1 and 5 after diagnosis.

### Diagnostics and treatment for urothelial cancer

The development of the tumor‐node‐metastasis (TNM) classification and staging system has been important for the standardization of diagnostics and treatment in cancers since 1958 [24]. Hematuria is the most common sign of UC and leads to cystoscopy [25]. Over the years, the diagnostic arsenal has increased to include ultrasound (US) and computed tomography (CT). A Finnish study defined the diagnostic periods: pre-CT and pre-US era (1964–1979), US era (1980–1988) and CT era (1989–1997) [26]. Detailed demographic and clinical data from bladder cancer patients recorded by the Swedish National Register of Urinary Bladder Cancer from 1997 to 2014 were reported [27, 28]; e.g. 74% of male and 68% of female tumors were non-muscle invasive, (2% data were missing) and the rest were muscle invasive (24% and 29%). According to this Register, there was a stage shift between periods 1997–2001 and 2007–2011 in clinical T categorization: Ta from 45% to 48%, T1 from 21.6% to 22.4%, and T2-T4 from 27% to 25% [29]. According to this source, between periods 1997–2001 and 2007–2011 intravesical treatment after transurethral resection for T1G2 and T1G3 tumors increased from 15% to 40% and from 30% to 50%, respectively; cystectomy for T2-T4 tumors increased from 30% to 40%.

In general, bladder cancer diagnostics, treatment and follow-up in Sweden and Finland have been uniform following national and European Association of Urology (EAU) guidelines [1, 25, 30, 31]. The urological association in Sweden was founded in 1950, in Finland 1954 and the Scandinavian Association of Urology had its first meeting in 1957 in Stockholm after its foundation in a Finnish sauna the previous year (http://www.nuf.nu/history/history.pdf).

## Results

The NORDCAN database includes 0.49 million male and 0.48 million female cancers for Finland, and 1.01 million male and 0.94 million female cancers for Sweden, excluding non-melanoma skin cancer, for years 1967 to 2016 (**S1 Table**). In Finland, male UCs numbered 27,667 compared to 9,212 female UCs; the related numbers for Sweden were 73,686 and 27,083.

**Fig 1:**
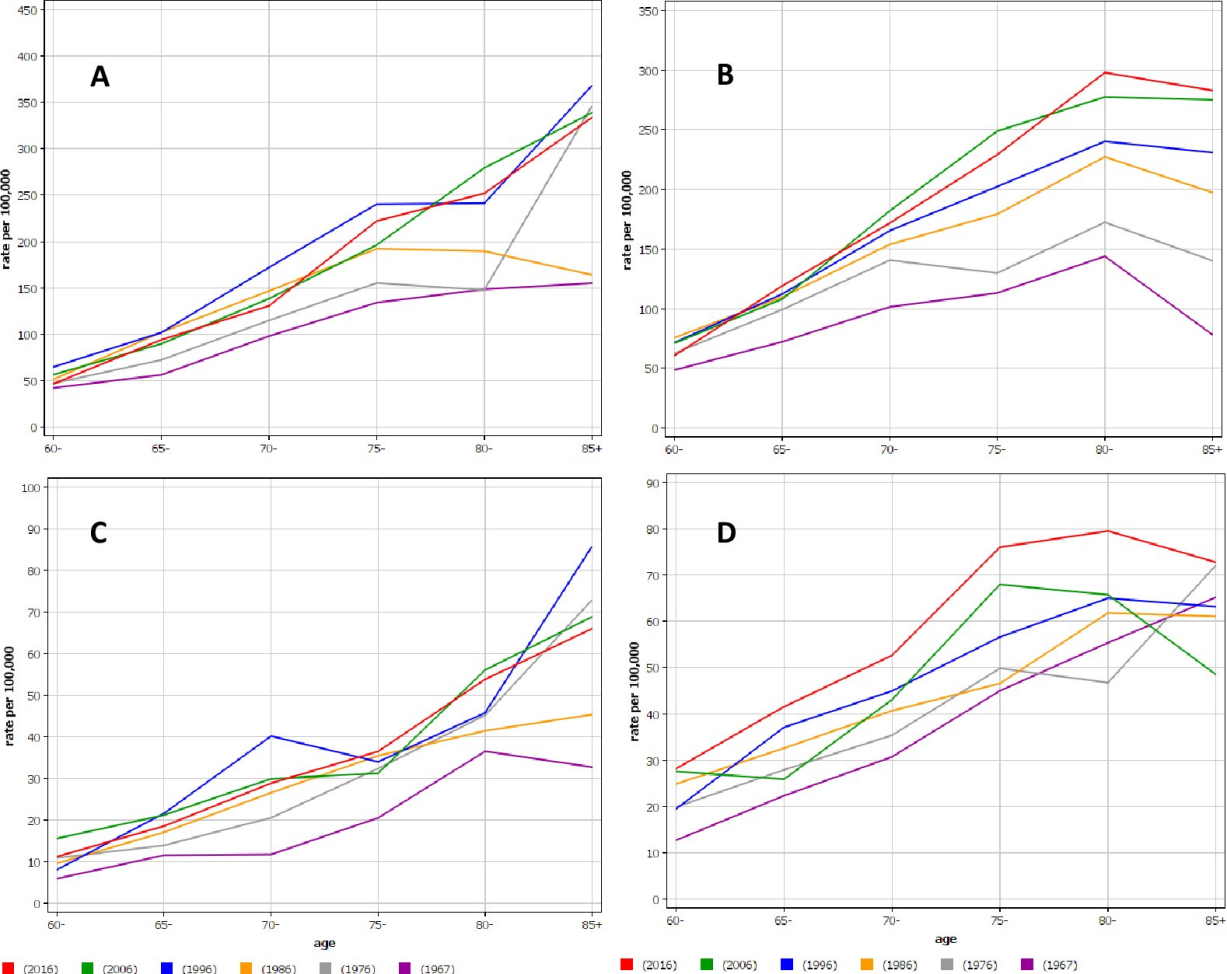
Annual age-specific incidence in urothelial cancer in Finnish (A) and Swedish (B) men and Finnish (C) and Swedish (D) women starting in year 1967 until year 1975 and from 1976 in 10-year intervals until year 2016. Note the different scales for the y-axes.

The median diagnostic ages were 72 years for Finnish and Swedish men, and 74 years for Finnish and 73 years for Swedish women. However, comparing the first and last 5-year periods the median diagnostic age increased with time, for Finnish men from 67 to 74 years, for Finnish women from 69 to 76 years, for Swedish men from 68 to 74 years, and women from 71 to 75 years. In **Fig 1** annual age-specific incidence is presented in 10-years intervals (the first interval 9 years from 1967 through 1975). For Finnish men (A) and women (C) the age-incidence graphs became steeper with time, implying ever higher incidence in older age. In Swedish men (B) the highest incidence was in age group 80–84 years but the difference to the incidence among 85+ year old became ever smaller with time. In Swedish women (D) the highest incidence was around age 80. The striking observation in these graphs was the vast time-dependent increase in incidence for the very old, more than 3-fold for Swedish men.

We calculated estimated annual percentage change (EAPC) and 95% confidence intervals (CIs) for the incidence trends in population older than 59 years from 1960 to 2016: Finnish men (N = 23,841) 1.45% [1.15;1.75], Swedish men (65,297) 1.52% [1.28;1.75], Finnish women (8282) 1.39% [1.10;1.68] and Swedish women (24,440) 1.15% [1.00;1.31]. For those aged over 79 years the increases were steeper, except for Swedish women: 2.14% [1.70;2.59], 2.30% [2.04;2.55], 1.83% [1.23;2.43] and 1.00% [0.79;1.22], respectively. In Finnish men aged 85+ EAPC was 2.37% [1.83;2.92] and in Swedish men it was 2.36% [2.06;2.67].

Relative 1-year and 5-year survival rates for UC for Finland and Sweden are shown in **Table 1.** The male 1-year survival increased constantly, from 71% (1967–1971) to 90% (2012–2016) for Finnish men and from 74% to 91% for Swedish men. For women the increases were from 70 to 86% (Finland) and from 71 to 85% (Sweden). The male 5-year survival increased from 45 to 79% (Finland) and from 55 to 77% (Sweden). For women the increases were from 48 to 74% and from 56 to 72%, respectively. In **Table 1**, the bolding between the periods indicates a significant increase in survival (i.e., 95% CIs were non-overlapping). Among men, large gains took place in the early period while for women no periodic increase was significant.

**Table 1:** 1-and 5-year age-standardized relative survival in percent [95% CI]. Finland and Sweden, age at diagnosis 0–89.

FINLAND	1967–1971		1972–1976		1977–1981		1982–1986		1987–1991		1992–1996		1997–2001		2002–2006		2007–2011		2012–2016	
Men, 1-year	71	[68;74]	** 76**	**[74;79]**	** 83**	**[81;85]**	85	[83;87]	87	[85;88]	89	[87;90]	89	[88;90]	89	[88;91]	89	[88;90]	90	[89;90]
Men, 5-year	45	[41;49]	** 51**	**[48;55]**	** 63**	**[60;66]**	67	[64;70]	71	[69;74]	75	[72;77]	75	[73;77]	76	[74;78]	78	[76;80]	79	[77;80]
**Difference** ^**a**^	**25**		**25**		**20**		**18**		**16**		**14**		**14**		**14**		**11**		**11**	
Women, 1-year	70	[65;75]	72	[68;76]	78	[75;82]	82	[79;85]	80	[77;83]	83	[81;86]	86	[83;88]	85	[83;88]	86	[84;88]	85	[83;87]
Women, 5-year	48	[42;55]	47	[43;52]	62	[57;67]	64	[59;68]	63	[60;67]	69	[66;72]	73	[69;76]	72	[69;76]	76	[72;79]	74	[71;76]
**Difference** ^**a**^	**22**		**25**		**16**		**18**		**17**		**14**		**13**		**13**		**10**		**11**	
**SWEDEN**																				
Men, 1-year	** 74**	**[73;76]**	** 78**	**[77;80]**	** 83**	**[82;84]**	85	[84;86]	87	[86;88]	88	[87;89]	88	[87;89]	88	[87;89]	89	[88;90]	91	[90;92]
Men, 5-year	** 55**	**[53;57]**	** 62**	**[60;64]**	** 68**	**[66;69]**	** 68**	**[67;70]**	** 72**	**[71;74]**	73	[72;75]	74	[73;76]	75	[73;76]	75	[74;77]	77	[76;78]
**Difference** ^**a**^	**19**		**16**		**15**		**17**		**15**		**15**		**14**		**13**		**14**		**14**	
Women, 1-year	71	[69;74]	76	[74;78]	78	[76;80]	81	[80;83]	82	[80;83]	85	[83;86]	83	[81;84]	84	[82;85]	85	[84;87]	85	[84;87]
Women, 5-year	56	[53;59]	61	[58;63]	62	[59;65]	66	[63;68]	67	[65;69]	69	[67;71]	69	[67;71]	70	[68;72]	72	[70;74]	72	[71;74]
**Difference** ^**a**^	**15**		**15**		**16**		**15**		**15**		**16**		**14**		**14**		**13**		**13**	

^**a**^ Difference between 1- and 5-year survival in percent units (% units)

**Fig 2:**
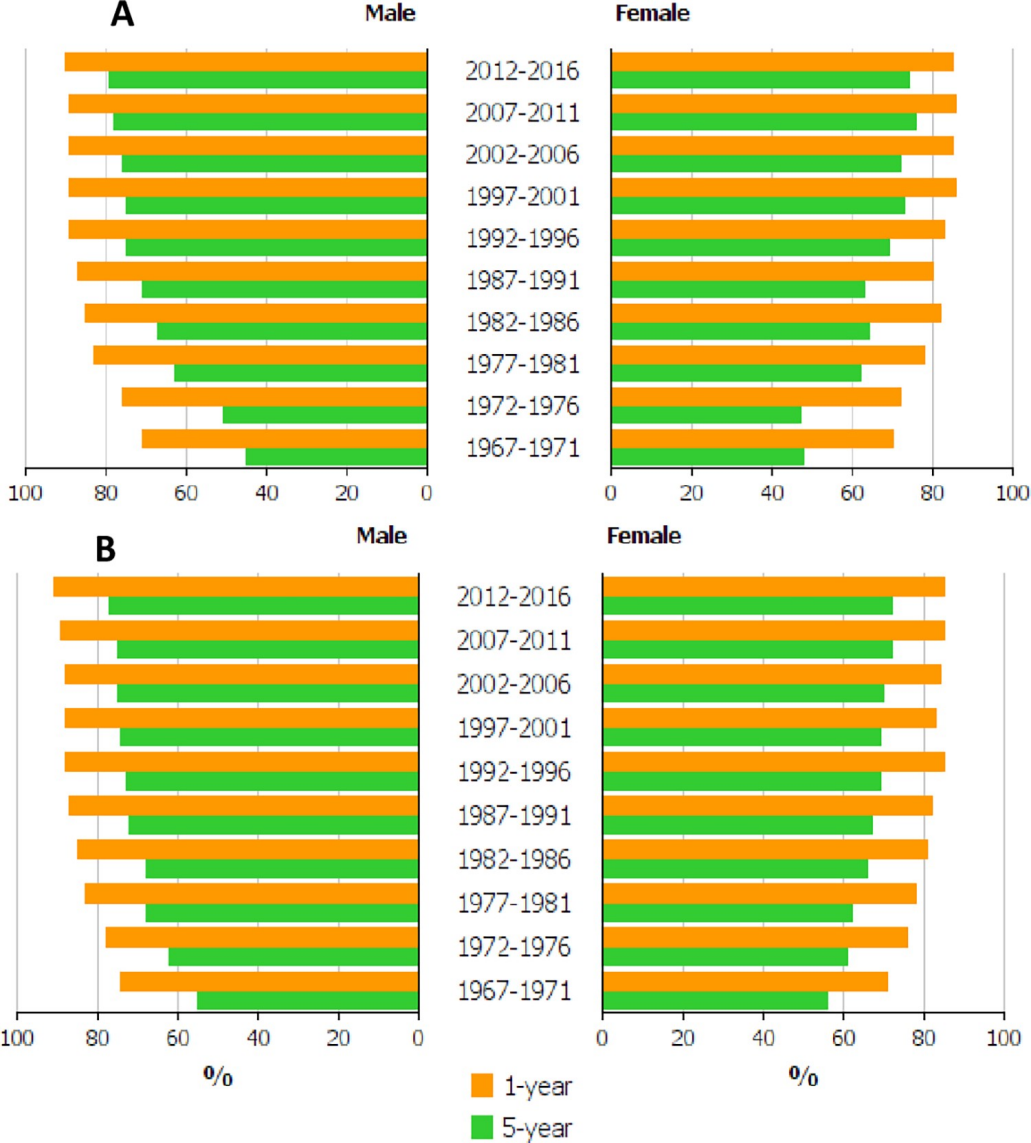
Relative 1-year (yellow bars) and 5-year (green bars) survival in urothelial cancer among Finnish (A) and Swedish (B) men and women.

**Table 1** shows the difference between 1- and 5-year survival in percent units (% units). In Finland, the difference decreased for men from 25 to 11% units, and for women from 22 to 11% units. In Sweden, the difference was initially lower for both sexes than in Finland but the changes were slower than in Finland. The survival data are plotted in **Fig 2**(A, Finland) and **2**(B, Sweden) illustrating the declining difference between the survival bars in Finland, opposite to Sweden.

**Fig 3:**
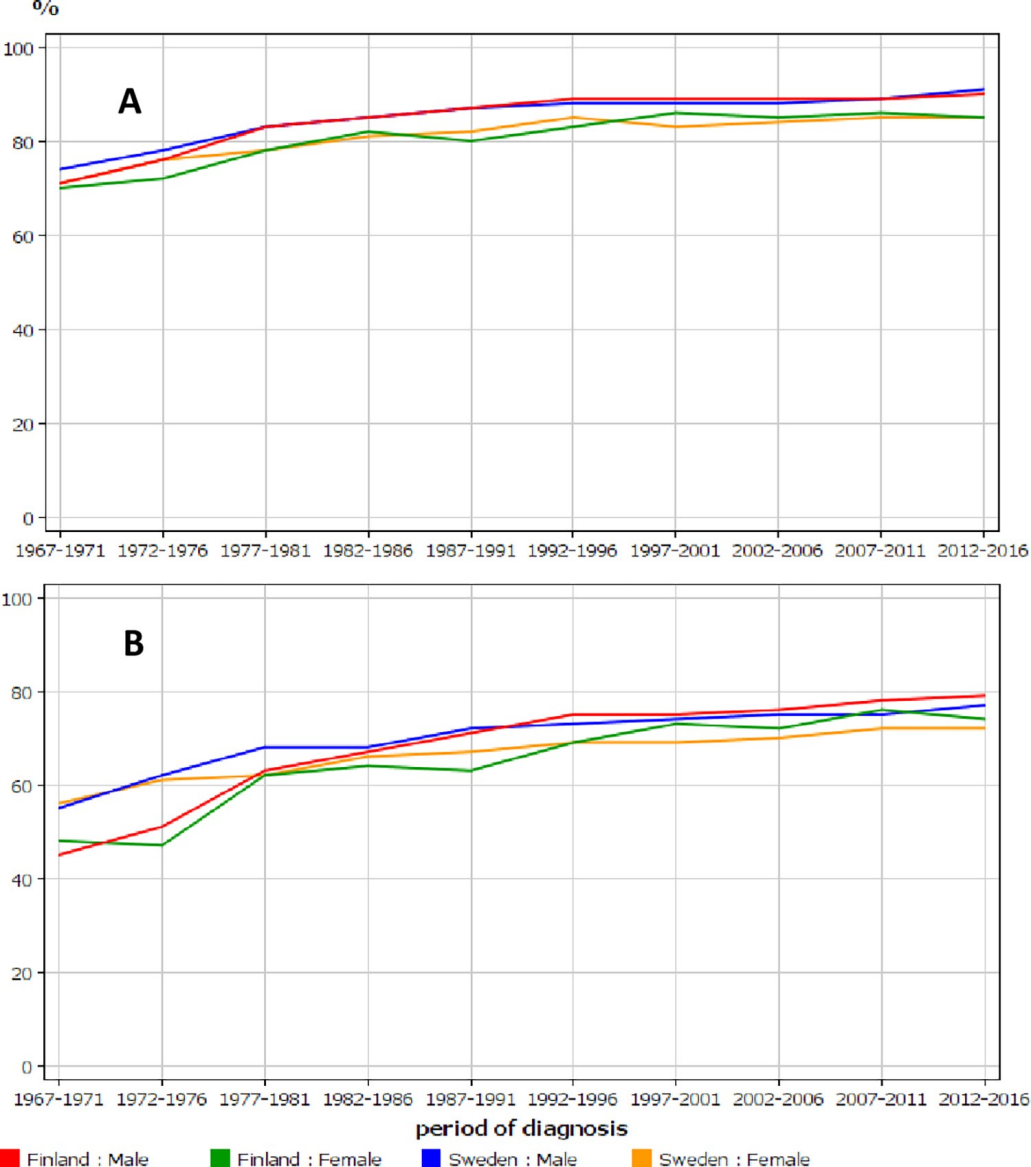
Relative 1-year (A) and 5-year (B) survival in urothelial cancer among Finnish and Swedish men and women in relation to time of diagnosis.

Trends in relative survival are shown for Finnish and Swedish men and women in **Fig 3.** The curves for 1-year survival are slightly higher for men than for women (Fig 3A) while in 5-year survival Finnish men did somewhat better and Swedish women somewhat worse towards of the end of the follow-up (Fig 3B).

**Fig 4:**
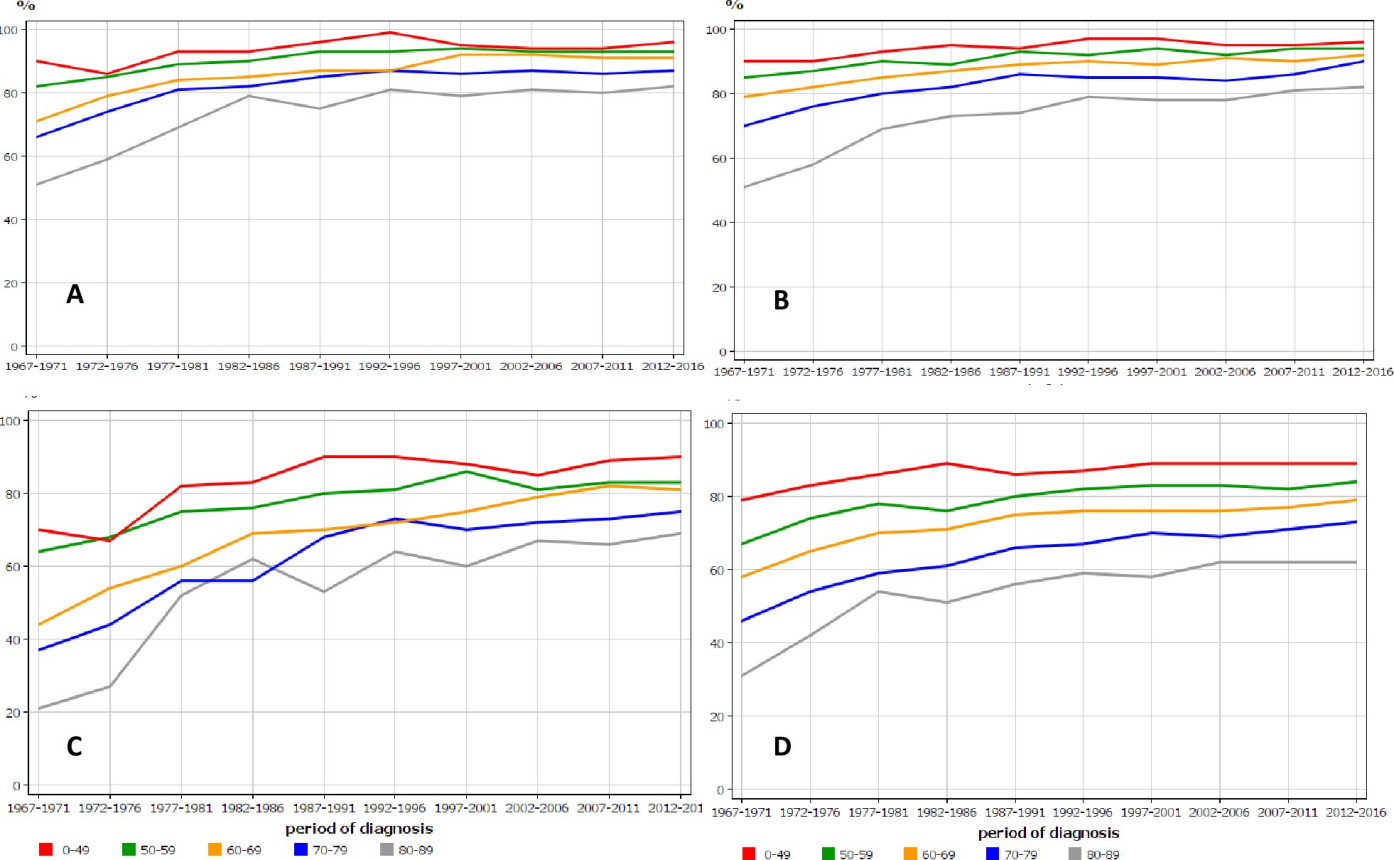
Age-specific 1-year relative survival for urothelial cancer in Finnish (A) and Swedish (B) men, and the related 5-year survival in the same male populations (C, D), in relation to time of diagnosis.

**Fig 5:**
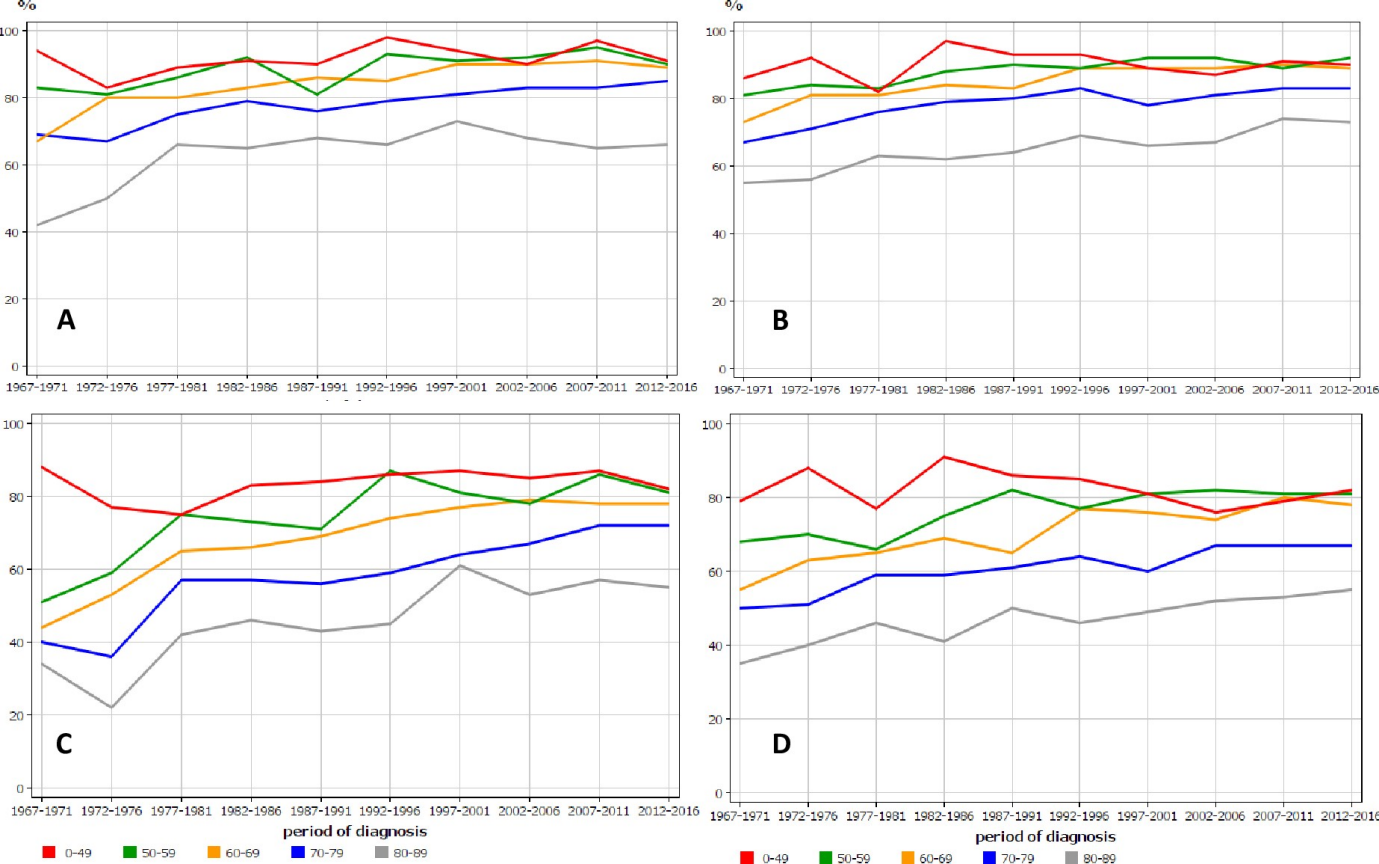
Age-specific 1-year relative survival for urothelial cancer in Finnish (A) and Swedish (B) women, and the related 5-year survival in the same female populations (C, D) in relation to time of diagnosis.

Age-specific 1- relative survival for UC in Finnish and Swedish men shows quite similar patterns of improving trends with time-dependent narrowing of differences between age groups (**Fig 4A and 4B**). More age group differences were observed for 5-year survival and old Swedish men lagged behind (**Fig 4C and 4D**). Age-specific data for women were similar but showed even a larger age group specific difference, and the two oldest age groups survived clearly worse than their younger counterparts in both countries **(Fig 5)**.

## Discussion

The novelty of the present study was the 50-year time span covered, while many cited Swedish survival studies covered a period from 1997 onwards (the Swedish National Register of Urinary Bladder Cancer was started then). Our **Table 1** and **Figs 2 and 3** show that little change (2–3% units increase) in 1-or 5-year survival took place in Sweden after 1997, while earlier the increases varied from 13 to 18% units. The other novel aspect was to compare survival differences between 1- and 5-year survival as an indication of survival gains at year 5, independent of 1-year survival.

The key findings of this study were the large shift of the diagnostic age and the peak incidence to older age and the main gains in 1-year survival were in the early part of the follow-up. In the country comparison, 5-year survival in Finland was initially almost 10% units below Sweden but passed Sweden towards the end. Age group specific 5-years survival revealed inferior prognoses for older and female patients, which remained to the end of the follow-up period.

The large shift towards higher diagnostic age is surprising in view of the advances in the imaging and diagnostic technologies, but, paradoxically, the increases may be the consequence of the technologies, which are able to find small lesions (**Fig 1**). A Swedish study based on the national bladder cancer register reported an increase in mean age from 71.7 years (1997–2001) to 72.5 years (2007–2011) [29]. In the US the Surveillance, Epidemiology and End Result (SEER) database the mean diagnostic age of bladder cancer increased by 1 year to 71.2 years in 10 years around year 2000 [32]. In the same database the stage-specific incidence of bladder cancer practically only increased for papillary noninvasive (Ta) tumors between 1988 and 2006 [33]. The trend was most dramatic among patients in the oldest age strata, supporting the notion of period-dependent wider use of imaging, easier access to flexible cystoscopy and generally more active diagnostics of older persons. For renal carcinoma there has been no such increase in median diagnostic ages [34]. Another reason for the period-dependent shifting of diagnostic age is of course aging of the population but Fig 1 showed that there was a marked shift to age group 85+ also in incidence, some 2-fold in Finnish men and women and 3-fold in Swedish men. The estimated annual percent changes for men aged 85+ from 1960 through 2016 were 2.37% [1.83;2.92] in Finland and 2.36% [2.06;2.67] in Sweden. Under-reporting of UC cases is an unlikely reason in view of a previous study [35].

One-year survival incorporates cured patients and patients alive with metastatic disease, while 5- year survival is nearly synonymous with patients who were cured with surgery with or without adjuvant therapies. While immunotherapy may be changing the situation currently, in the period of the present study few patients with metastatic disease survived more than 5 years. The increase in 1-year survival ranged between 10 and 16% units in the first 25-year period and slowed to 0–3% unit in the subsequent 25-year period (**Table 1**). Of course, the reason is that at or close to 90% survival, any gains are hard to earn. As most fatalities in the first year after diagnosis would be due to muscle-invasive tumors with distal metastases, the improvements in treatment and/or early detection during the first 25-year period helped to achieve large gains in 1-year survival. Also 5-year survival increased independent of 1-years gains as shown by the decrease in the difference between 1- and 5-years survival in all patients except Swedish women. A Swedish study covering a period from 1997 to 2011 reported on clinical treatment and stage-specific survival, concluding in its title: ‘No difference in relative survival over time despite more aggressive treatment’ [29]. Our result regarding the indicated period were in line with these findings. The ‘more aggressive’ therapies have included more frequent second resections, increased use of intravesical Bacillus Calmette-Guerin or chemotherapy instillations and introduction of neoadjuvant chemotherapies [13]. In the 1980s cisplatin chemotherapy was introduced in advanced bladder cancer or as neoadjuvant therapy might have contributed to the increased 1-year survival [36].

Although survival in Finland and Sweden in the last 1-year period 2012–16 was almost identical (men 90/91%, women 85%), 5-year survival was somewhat more favorable in Finland (men 79%, women 74%) than in Sweden (men 77%, women 72%). The different development was illustrated by the difference between 1- and 5-year survival, which markedly declined in Finland but marginally improved in Sweden. The main reason for the difference is the low 5-year survival in Finland in the early follow-up period.

Smoking increases complications in bladder cancer treatment and survival, but smoking prevalence is an unlikely explanation for men because Swedish men have had the lowest smoking frequency in Europe [37–40]. As an opposite trend, use of oral tobacco (snus) has increased among Sweden men (1988 19%, 2004 27%), and a combined daily use of either form of tobacco product has remained constant in Sweden (1988 43%, 2004 40%) [37, 41]. While the use of oral tobacco has also increase among Swedish women (1988 1%, 2004 4%) and Finnish men (1988 nil, 2004 3%) its use in these groups is still relatively uncommon and not likely to affect our results [37]. In 1995, both countries joined the European Union illegalizing the sales of oral tobacco, yet Sweden negotiated an exempt in this regard. In women daily smoking has been more common in Sweden (1988 32%, 2004 20%) than in Finland (1988 20%, 2004 21%) [37]. Snus may increase the risk and mortality of some cancers but only indirect data are available on its possible influence on survival in bladder cancer [41, 42]. However, trends in smoking and the use of oral tobacco might explain why Swedish males and females lost their lead in 5-year survival to their Finnish counterparts. Another difference in the Finnish and Swedish populations is the high prevalence of immigrants in Sweden; some 15% of cancers are diagnosed in persons who were born outside Sweden [43]. Nevertheless, bladder cancer incidence is overall below the risk in native Swedes [44].

Survival graphs revealed disparities between age groups, which however narrowed in the course of time (**Figs 4 and 5**). The differences in 1-year survival were rather small in the last period but remained wide in 5-year survival. The reasons may be differential treatment and less regular follow-up. For example, intravesical therapy was less frequently used for old patients diagnosed with in situ bladder cancer in Sweden [45].

The worse survival for women was another disparity. A previous study reported that the gender difference was limited to higher stage muscle-invasive tumors, for which intravesical instillation therapy was less frequently used than for men [27]. Similar differences were also reported in another study on T1 bladders cancer, which also recognized a two year higher diagnostic age in women (76 years) [46]. The common explanations for gender differences include women’s less concern about episodes of hematuria as menstruation has been a normal part of their early lives. In contrary, most males will want this extraordinary event promptly investigated. Another difference is that females tend to seek investigations from their gynecological doctor and typical gynecological investigations may not include cystoscopy. Cystoscopy on the other hand will be the immediate investigation in the urological side. Delays in diagnosis have been shown to correlate in worse survival in bladder cancer [47].

The strengths of the study are that we have high-level diagnostic data from two countries with practically free medical care, thus offering a rare opportunity to view historical ‘real world’ medical experience in these cancers. The weaknesses are basically of two kinds, the study is ecological and no individual level treatment or care data were available [48]. Lack of stage data in the NORDCAN database does not allow inclusion of this variable. However, a Swedish study based on National Registry of Urinary Bladder Cancer from 1997 to 2016, showed that survival improved in all stage categories, irrespective of age and gender [13]. Thus, at least in the last period the minor improvements in survival benefited various stages in parallel.

## Conclusions

These ‘real world’ data showed that the main survival gains were achieved in Finland and Sweden until the early 1990s, and the small improvements until 2012–2016 helped to attain the 90% mark for male 1-years survival and 85% mark for female survival. While 5-year survival remained more than 10% unit below 1-year survival, it was worrisome that after the early 1990s the gap (difference) between 1-and 5-years survival narrowed only modestly, if at all. Further improvements in survival call for control of smoking, assessment of the role of snus in Sweden [41], emphasis on early diagnosis, including prompt cystoscopy and imaging investigations in hematuria, and upfront curative treatment with surgery and adjuvant therapies, combined with awareness of high relapse rates in treated bladder cancer patients requiring regular cystoscopy follow up.

## Supporting information

S1 Table Numbers of patients with urothelial cancer and with any cancer in Finland and Sweden 1967 to 2015. (PPTX)

S2 Table STROBE statement—checklist of items that should be included in reports of observational studies. (DOCX)

## References

[1] Roupret M, Babjuk M, Comperat E, Zigeuner R, Sylvester RJ, Burger M, et al. European Association of Urology Guidelines on Upper Urinary Tract Urothelial Carcinoma: 2017 Update. Eur Urol. 2018;73(1):111–22. Epub 2017/09/05. doi: 10.1016/j.eururo.2017.07.036 . 28867446

[2] Teoh JY, Huang J, Ko WY, Lok V, Choi P, Ng CF, et al. Global Trends of Bladder Cancer Incidence and Mortality, and Their Associations with Tobacco Use and Gross Domestic Product Per Capita. Eur Urol. 2020;78(6):893–906. Epub 2020/09/26. doi: 10.1016/j.eururo.2020.09.006 . 32972792

[3] Cai Q, Chen Y, Xin S, Zhang D, Pan J, Xie Z, et al. Temporal trends of bladder cancer incidence and mortality from 1990 to 2016 and projections to 2030. Transl Androl Urol. 2020;9(2):153–65. Epub 2020/05/19. doi: 10.21037/tau.2020.02.24 ; PubMed Central PMCID: PMC7215039. 32420122 PMC7215039

[4] Ebrahimi H, Amini E, Pishgar F, Moghaddam SS, Nabavizadeh B, Rostamabadi Y, et al. Global, Regional and National Burden of Bladder Cancer, 1990 to 2016: Results from the GBD Study 2016. J Urol. 2019;201(5):893–901. Epub 2019/01/25. doi: 10.1097/JU.0000000000000025 . 30676477

[5] Brown KF, Rumgay H, Dunlop C, Ryan M, Quartly F, Cox A, et al. The fraction of cancer attributable to modifiable risk factors in England, Wales, Scotland, Northern Ireland, and the United Kingdom in 2015. Br J Cancer. 2018;118(8):1130–41. Epub 2018/03/24. doi: 10.1038/s41416-018-0029-6 ; PubMed Central PMCID: PMC5931106. 29567982 PMC5931106

[6] Kohlmann W, Gruber SB. Lynch Syndrome. In: Adam MP, Ardinger HH, Pagon RA, Wallace SE, Bean LJH, Stephens K, et al., editors. GeneReviews((R)). Seattle (WA): University of Washington, Seattle University of Washington, Seattle. GeneReviews is a registered trademark of the University of Washington, Seattle. All rights reserved.; 1993. doi: 10.1093/ajh/6.9.794

[7] Yu H, Hemminki K. Genetic epidemiology of colorectal cancer and associated cancers. Mutagenesis. 2020;35:207–19. Epub 2019/08/20. doi: 10.1093/mutage/gez022 . 31424514

[8] Aragon-Ching JB, Nizam A, Henson DE. Carcinomas of the Renal Pelvis, Ureters, and Urinary Bladder Share a Carcinogenic Field as Revealed in Epidemiological Analysis of Tumor Registry Data. Clinical genitourinary cancer. 2019;17(6):436–42. Epub 2019/08/26. doi: 10.1016/j.clgc.2019.07.011 . 31445851

[9] Liu X, Hemminki K, Forsti A, Sundquist K, Sundquist J, Ji J. Cancer risk in patients with type 2 diabetes mellitus and their relatives. Int J Cancer. 2015. Epub 2015/01/22. doi: 10.1002/ijc.29440 25604005

[10] Møller P, Seppälä TT, Bernstein I, Holinski-Feder E, Sala P, Gareth Evans D, et al. Cancer risk and survival in path_MMR carriers by gene and gender up to 75 years of age: a report from the Prospective Lynch Syndrome Database. Gut. 2018;67(7):1306–16. Epub 2017/07/30. doi: 10.1136/gutjnl-2017-314057 ; PubMed Central PMCID: PMC6031262. 28754778 PMC6031262

[11] Wischhusen JW, Ukaegbu C, Dhingra TG, Uno H, Kastrinos F, Syngal S, et al. Clinical Factors Associated with Urinary Tract Cancer in Individuals with Lynch Syndrome. Cancer Epidemiol Biomarkers Prev. 2020;29(1):193–9. Epub 2019/10/17. doi: 10.1158/1055-9965.EPI-19-0213 ; PubMed Central PMCID: PMC6954282. 31615790 PMC6954282

[12] Nilsson S, Ragnhammar P, Glimelius B, Nygren P. A systematic overview of chemotherapy effects in urothelial bladder cancer. Acta Oncol. 2001;40(2–3):371–90. Epub 2001/07/10. doi: 10.1080/02841860151116466 . 11441942

[13] Malmstrom PU, Gardmark T, Sherif A, Strock V, Hosseini-Aliabad A, Jahnson S, et al. Incidence, survival and mortality trends of bladder cancer in Sweden 1997–2016. Scandinavian journal of urology. 2019;53(4):193–9. Epub 2019/07/03. doi: 10.1080/21681805.2019.1632380 . 31262208

[14] Feldman AS, Kulkarni GS, Bivalacqua TJ, Black PC, Delacroix S, Lerner SP, et al. Surgical challenges and considerations in Tri-modal therapy for muscle invasive bladder cancer. Urol Oncol. 2021. Epub 2021/03/02. doi: 10.1016/j.urolonc.2021.01.013 33642229

[15] De Angelis R, Sant M, Coleman MP, Francisci S, Baili P, Pierannunzio D, et al. Cancer survival in Europe 1999–2007 by country and age: results of EUROCARE—5-a population-based study. Lancet Oncol. 2014;15(1):23–34. Epub 2013/12/10. doi: 10.1016/S1470-2045(13)70546-1 . 24314615

[16] Engholm G, Hakulinen T, Gislum M, Tryggvadóttir L, Klint A, Bray F, et al. Trends in the survival of patients diagnosed with kidney or urinary bladder cancer in the Nordic countries 1964–2003 followed up to the end of 2006. Acta Oncol. 2010;49(5):655–64. Epub 2010/02/17. doi: 10.3109/02841860903575299 . 20156116

[17] Hussain SK, Lenner P, Sundquist J, Hemminki K. Influence of education level on cancer survival in Sweden. Ann Oncol. 2008;19:156–62. doi: 10.1093/annonc/mdm413 . 17785761

[18] Marcos-Gragera R, Mallone S, Kiemeney LA, Vilardell L, Malats N, Allory Y, et al. Urinary tract cancer survival in Europe 1999–2007: Results of the population-based study EUROCARE-5. Eur J Cancer. 2015;51(15):2217–30. Epub 2015/10/01. doi: 10.1016/j.ejca.2015.07.028 . 26421824

[19] Kim M, Jeong CW, Kwak C, Kim HH, Ku JH. Are urothelial carcinomas of the upper urinary tract a distinct entity from urothelial carcinomas of the urinary bladder? Behavior of urothelial carcinoma after radical surgery with respect to anatomical location: a case control study. BMC Cancer. 2015;15:149. Epub 2015/04/18. doi: 10.1186/s12885-015-1161-9 ; PubMed Central PMCID: PMC4369352. 25886012 PMC4369352

[20] Welch HG, Kramer BS, Black WC. Epidemiologic Signatures in Cancer. N Engl J Med. 2019;381(14):1378–86. Epub 2019/10/03. doi: 10.1056/NEJMsr1905447 . 31577882

[21] Engholm G, Ferlay J, Christensen N, Bray F, Gjerstorff ML, Klint A, et al. NORDCAN—a Nordic tool for cancer information, planning, quality control and research. Acta Oncol. 2010;49(5):725–36. Epub 2010/05/25. doi: 10.3109/02841861003782017 . 20491528

[22] Storm HH, Klint A, Tryggvadóttir L, Gislum M, Engholm G, Bray F, et al. Trends in the survival of patients diagnosed with malignant neoplasms of lymphoid, haematopoietic, and related tissue in the Nordic countries 1964–2003 followed up to the end of 2006. Acta Oncol. 2010;49(5):694–712. Epub 2010/05/25. doi: 10.3109/02841861003631495 . 20491526

[23] Engholm G, Gislum M, Bray F, Hakulinen T. Trends in the survival of patients diagnosed with cancer in the Nordic countries 1964–2003 followed up to the end of 2006. Material and methods. Acta Oncol. 2010;49(5):545–60. Epub 2010/05/25. doi: 10.3109/02841861003739322 . 20491523

[24] Greene FL, Sobin LH. The staging of cancer: a retrospective and prospective appraisal. CA Cancer J Clin. 2008;58(3):180–90. Epub 2008/05/08. doi: 10.3322/CA.2008.0001 . 18460593

[25] Oosterlinck W, Lobel B, Jakse G, Malmström PU, Stöckle M, Sternberg C. Guidelines on bladder cancer. Eur Urol. 2002;41(2):105–12. Epub 2002/06/21. doi: 10.1016/s0302-2838(01)00026-4 . 12074395

[26] Sunela KL, Lehtinen ET, Kataja MJ, Kujala PM, Soimakallio S, Kellokumpu-Lehtinen PL. Development of renal cell carcinoma (RCC) diagnostics and impact on prognosis. BJU Int. 2014;113(2):228–35. Epub 2013/07/31. doi: 10.1111/bju.12242 . 23890347

[27] Radkiewicz C, Edgren G, Johansson ALV, Jahnson S, Häggström C, Akre O, et al. Sex Differences in Urothelial Bladder Cancer Survival. Clinical genitourinary cancer. 2020;18(1):26–34.e6. Epub 2019/12/04. doi: 10.1016/j.clgc.2019.10.020 . 31787542

[28] Häggström C, Liedberg F, Hagberg O, Aljabery F, Ströck V, Hosseini A, et al. Cohort profile: The Swedish National Register of Urinary Bladder Cancer (SNRUBC) and the Bladder Cancer Data Base Sweden (BladderBaSe). BMJ Open. 2017;7(9):e016606. Epub 2017/10/01. doi: 10.1136/bmjopen-2017-016606 ; PubMed Central PMCID: PMC5623498. 28963292 PMC5623498

[29] Jahnson S, Hosseini Aliabad A, Holmäng S, Jancke G, Liedberg F, Ljungberg B, et al. Swedish National Registry of Urinary Bladder Cancer: No difference in relative survival over time despite more aggressive treatment. Scandinavian journal of urology. 2016;50(1):14–20. Epub 2015/09/19. doi: 10.3109/21681805.2015.1085089 . 26382667

[30] Babjuk M, Burger M, Compérat EM, Gontero P, Mostafid AH, Palou J, et al. European Association of Urology Guidelines on Non-muscle-invasive Bladder Cancer (TaT1 and Carcinoma In Situ) - 2019 Update. Eur Urol. 2019;76(5):639–57. Epub 2019/08/25. doi: 10.1016/j.eururo.2019.08.016 . 31443960

[31] Witjes JA, Bruins HM, Cathomas R, Compérat EM, Cowan NC, Gakis G, et al. European Association of Urology Guidelines on Muscle-invasive and Metastatic Bladder Cancer: Summary of the 2020 Guidelines. Eur Urol. 2021;79(1):82–104. Epub 2020/05/04. doi: 10.1016/j.eururo.2020.03.055 . 32360052

[32] Zang Y, Li X, Cheng Y, Qi F, Yang N. An overview of patients with urothelial bladder cancer over the past two decades: a Surveillance, Epidemiology, and End Results (SEER) study. Ann Transl Med. 2020;8(23):1587. Epub 2021/01/14. doi: 10.21037/atm-20-2108 ; PubMed Central PMCID: PMC7791213. 33437786 PMC7791213

[33] Nielsen ME, Smith AB, Meyer AM, Kuo TM, Tyree S, Kim WY, et al. Trends in stage-specific incidence rates for urothelial carcinoma of the bladder in the United States: 1988 to 2006. Cancer. 2014;120(1):86–95. Epub 2013/10/15. doi: 10.1002/cncr.28397 ; PubMed Central PMCID: PMC3964001. 24122346 PMC3964001

[34] Thorstenson A, Bergman M, Scherman-Plogell AH, Hosseinnia S, Ljungberg B, Adolfsson J, et al. Tumour characteristics and surgical treatment of renal cell carcinoma in Sweden 2005–2010: a population-based study from the national Swedish kidney cancer register. Scandinavian journal of urology. 2014;48(3):231–8. Epub 2014/03/29. doi: 10.3109/21681805.2013.864698 . 24666102

[35] Ji J, Sundquist K, Sundquist J, Hemminki K. Comparability of cancer identification among Death Registry, Cancer Registry and Hospital Discharge Registry. Int J Cancer. 2012;131:2085–93. Epub 2012/02/07. doi: 10.1002/ijc.27462 . 22307919

[36] Malmström PU, Rintala E, Wahlqvist R, Hellström P, Hellsten S, Hannisdal E. Five-year followup of a prospective trial of radical cystectomy and neoadjuvant chemotherapy: Nordic Cystectomy Trial I. The Nordic Cooperative Bladder Cancer Study Group. J Urol. 1996;155(6):1903–6. Epub 1996/06/01. 8618283

[37] Patja K, Hakala SM, Boström G, Nordgren P, Haglund M. Trends of tobacco use in Sweden and Finland: do differences in tobacco policy relate to tobacco use? Scand J Public Health. 2009;37(2):153–60. Epub 2009/01/24. doi: 10.1177/1403494808100277 . 19164430

[38] Tellini R, Mari A, Muto G, Cacciamani GE, Ferro M, Stangl-Kremser J, et al. Impact of Smoking Habit on Perioperative Morbidity in Patients Treated with Radical Cystectomy for Urothelial Bladder Cancer: A Systematic Review and Meta-analysis. European urology oncology. 2020. Epub 2020/11/09. doi: 10.1016/j.euo.2020.10.006 33160975

[39] Rink M, Furberg H, Zabor EC, Xylinas E, Babjuk M, Pycha A, et al. Impact of smoking and smoking cessation on oncologic outcomes in primary non-muscle-invasive bladder cancer. Eur Urol. 2013;63(4):724–32. Epub 2012/08/29. doi: 10.1016/j.eururo.2012.08.025 ; PubMed Central PMCID: PMC3969986. 22925575 PMC3969986

[40] Hemminki K, Försti A, Hemminki A, Ljungberg B, Hemminki O. Incidence trends in lung and bladder cancers in the Nordic Countries before and after the smoking epidemic. Eur J Cancer Prev. 2021. Epub 2021/06/03. doi: 10.1097/cej.0000000000000694 34074862

[41] Hemminki K, Försti A, Hemminki A, Ljungberg B, Hemminki O. Incidence trends in bladder and lung cancers between Denmark, Finland and Sweden may implicate oral tobacco (snuff/snus) as a possible risk factor. BMC Cancer. 2021;21(1):604. Epub 2021/05/27. doi: 10.1186/s12885-021-08371-w ; PubMed Central PMCID: PMC8152093. 34034676 PMC8152093

[42] Inoue-Choi M, Shiels MS, McNeel TS, Graubard BI, Hatsukami D, Freedman ND. Contemporary Associations of Exclusive Cigarette, Cigar, Pipe, and Smokeless Tobacco Use With Overall and Cause-Specific Mortality in the United States. JNCI Cancer Spectr. 2019;3(3):pkz036. Epub 2019/07/20. doi: 10.1093/jncics/pkz036 ; PubMed Central PMCID: PMC6620791. 31321380 PMC6620791

[43] Hemminki K, Forsti A, Khyatti M, Anwar WA, Mousavi M. Cancer in immigrants as a pointer to the causes of cancer. European journal of public health. 2014;24 Suppl 1:64–71. Epub 2014/08/12. doi: 10.1093/eurpub/cku102 . 25108000

[44] Mousavi SM, Sundquist J, Hemminki K. Risk of transitional-cell carcinoma of the bladder in first- and second-generation immigrants to Sweden. Eur J Cancer Prev. 2010;19(4):275–9. Epub 2010/06/11. doi: 10.1097/cej.0b013e3283387728 . 20535860

[45] Jancke G, Liedberg F, Aljabery F, Sherif A, Ströck V, Malmström PU, et al. Intravesical instillations and cancer-specific survival in patients with primary carcinoma in situ of the urinary bladder. Scandinavian journal of urology. 2017;51(2):124–9. Epub 2017/03/30. doi: 10.1080/21681805.2017.1298156 . 28351206

[46] Sjöström C, Thorstenson A, Ströck V, Hosseini-Aliabad A, Aljabery F, Liedberg F, et al. Treatment according to guidelines may bridge the gender gap in outcome for patients with stage T1 urinary bladder cancer. Scandinavian journal of urology. 2018;52(3):186–93. Epub 2018/04/21. doi: 10.1080/21681805.2018.1462254 . 29676191

[47] Fahmy NM, Mahmud S, Aprikian AG. Delay in the surgical treatment of bladder cancer and survival: systematic review of the literature. Eur Urol. 2006;50(6):1176–82. Epub 2006/07/19. doi: 10.1016/j.eururo.2006.05.046 . 16846680

[48] Hemminki K, Försti A, Hemminki A, Ljungberg B, Hemminki O. Progress in survival in renal cell carcinoma through 50 years evaluated in Finland and Sweden. PLoS One. 2021;16(6):e0253236. Epub 2021/06/23. doi: 10.1371/journal.pone.0253236 ; PubMed Central PMCID: PMC8219161 interests: AH is shareholder in Targovax ASA. AH is also employee and shareholder in TILT Biotherapeutics Ltd. Other authors declared no conflict of interest. This does not alter our adherence to PLOS ONE policies on sharing data and materials. There are no patents, products in development or marketed products associated with this research to declare. 34157049 PMC8219161

