# Supplementary figures and images for: Survival in bladder and upper urinary tract cancers in Finland and Sweden through 50 years
Source: PLoS One. 2022 Jan 4;17(1):e0261124. doi: 10.1371/journal.pone.0261124 (PMC8726478; doi:10.1371/journal.pone.0261124)

## Slide 1
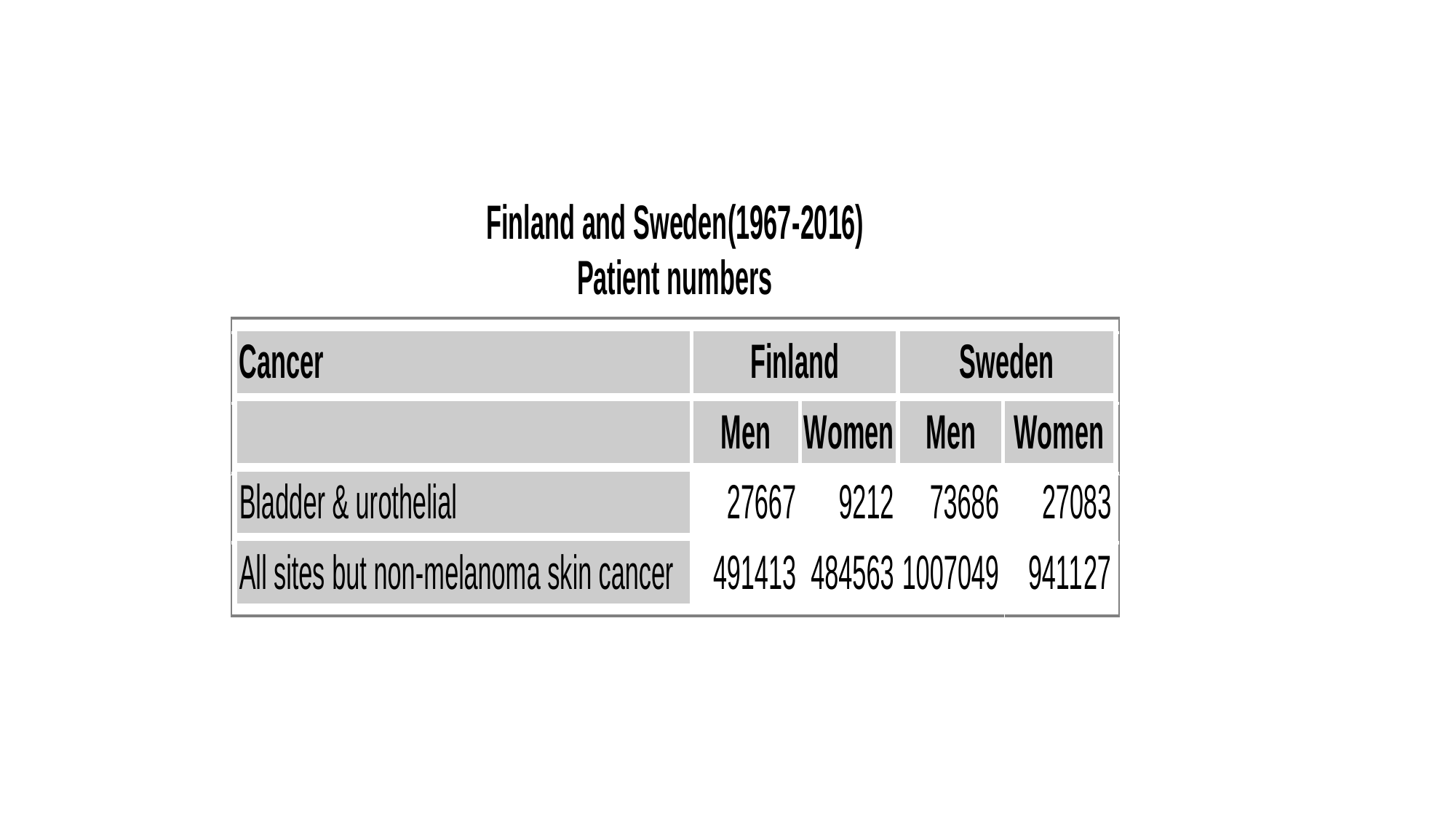

Supplement: S1 Table — (PPTX) [file pone.0261124.s001.pptx]
